# Circulating microRNA-101 as a potential biomarker for hepatitis B virus-related hepatocellular carcinoma

**DOI:** 10.3892/ol.2013.1638

**Published:** 2013-10-21

**Authors:** YU FU, XUFU WEI, CHENGYONG TANG, JIANPING LI, RUI LIU, AI SHEN, ZHONGJUN WU

**Affiliations:** 1 Department of Hepatobiliary Surgery, The First Affiliated Hospital of Chongqing Medical University, Yuzhong, Chongqing 400016, P.R. China; 2 Department of Clinical Pharmacology, The First Affiliated Hospital of Chongqing Medical University, Yuzhong, Chongqing 400016, P.R. China; 3 Department of Histology and Embryology, Chongqing Medical University, Yuzhong, Chongqing 400016, P.R. China

**Keywords:** miR-101, serum, monitor, HBV-related HCC

## Abstract

Circulating microRNAs (miRNAs) are emerging as promising biomarkers for cancer; however, the significance of circulating miRNAs in hepatitis B virus (HBV)-related hepatocellular carcinoma (HCC) remains largely unknown. Based on our prior observations that miRNA-101 (miR-101) is downregulated by HBV and induces epigenetic modification, we sought to test whether circulating miR-101 may serve as a potential biomarker for HCC. The expression of miR-101 in HCCs and serum was evaluated by real-time polymerase chain reaction. Tissue and serum miR-101 levels were assessed in samples from patients with HBV-related HCC and healthy controls. A potential correlation was also evaluated between miR-101 expression and the clinicopathological features and prognosis of HCC patients. miR-101 was downregulated in HBV-related HCC tissues compared with adjacent noncancerous tissues. Furthermore, the miR-101 levels in these tissues from HCC patients were significantly lower than those in tissues from control subjects. Notably, serum miR-101 levels were found to have an inverse correlation with tissue miR-101 expression levels. The expression of serum miR-101 in patients with HBV-related HCC was significantly higher than that in the healthy controls, and this increase correlated with hepatitis B surface antigen positivity, HBV DNA levels and tumor size. These results indicate that different factors govern the levels of miR-101 in the tissue and serum of HCC patients. Given the marked and consistent increase in serum miR-101 levels in HCC patients, circulating miR-101 may serve as a promising biochemical marker for monitoring the progression of tumor development in HBV-related HCC.

## Introduction

Hepatocellular carcinoma (HCC), the most common primary liver cancer, is a highly lethal disease that causes ~700,000 mortalities worldwide each year (1). It has been reported that as many as 80% of HCC cases can be attributed to chronic hepatitis B virus (HBV) infection (2). Currently, there are no biomarkers for the early detection of HCC, and the majority of patients with HCC are diagnosed at advanced stages, which are associated with a poor prognosis and low survival rates due to a lack of curative treatment options. A common approach used for screening HCC in a high risk-population is to examine serum tumor markers, such as α-fetoprotein (AFP). However, the sensitivity and specificity of serum AFP levels for HCC have been reported to range from 39–64% and 76–91%, respectively, indicating that elevated serum AFP levels are not a sufficient indicator of HCC (3,4). Thus, it is critical to identify novel biochemical markers for the early detection of HCC.

microRNAs (miRNAs) are small non-coding RNAs that post-transcriptionally regulate gene expression, predominantly through imperfect base pairing with the 3′-untranslated region of target mRNAs (5). miRNAs affect a broad range of biological functions including development, apoptosis, proliferation and differentiation (6–8). Dysregulation of miRNAs is also implicated in various diseases, including cancer. There is evidence that clearly demonstrates that, apart from genetic and epigenetic abnormalities, the dysregulation of miRNAs may also contribute to the aberrant activation of oncogenes and the inactivation of tumor suppressor genes in human carcinogenesis (9,10). Aberrant expression of miRNAs has been widely reported in human cancers with both up- and downregulation detected in HCC tumor tissue relative to the corresponding normal tissue (11–13). miRNAs are notably stable in blood, and their expression patterns appear to be tissue-specific. These characteristics make circulating miRNAs good candidates for noninvasive testing for cancer. Circulating miRNAs have been suggested as diagnostic markers for various types of cancer (14–19). Previously, we reported that miRNA-101 (miR-101) is downregulated by the HBV X protein (20). However, the applicability of circulating miR-101 in the diagnosis of HBV-related HCC has not been explored.

## Materials and methods

### Serum and tissue specimens

Serum and tissues (paired tissue specimens from HBV-related HCC tissues and adjacent noncancerous hepatic tissues) were obtained from patients undergoing surgical HCC resection. The specimens were collected at the Hepatobiliary Surgery Department of the First Affiliated Hospital of Chongqing Medical University, Chongqing, China. As a control, serum was also collected from healthy volunteers. Volunteers had not been diagnosed previously with any type of cancer, based on self-reporting. All participants signed informed consent for the use of their blood samples prior to recruitment. This study was approved by the Ethics Committee of The First Affiliated Hospital of Chongqing Medical University.

### RNA extraction

Total RNA was extracted from serum using TRIzol reagent (Invitrogen, Carlsbad, CA, USA) according to the instructions provided by the manufacturer. The purity of the isolated RNA was determined by OD260/280-reading using a NanodropND-2000 spectrophotometer (Thermo Scientific, Worcester, MA, USA).

### Real-time quantitative reverse transcription-polymerase chain reaction (qPCR) for miRNA expression

Reverse transcription was performed using the M-MLV Reverse Transcription system (Promega Corporation, Madison, WI, USA). U6 RNA was used as an internal control for the miRNA. The primers used for stem-loop reverse-transcription PCR for miR-101 were purchased from RiboBio Co., Ltd. (Guangzhou, China). qPCR was performed using a standard SYBR-Green PCR kit protocol for a StepOne Plus system (Applied Biosystems, Foster City, CA, USA). Finally, relative expression was calculated using the comparative Ct method and normalized to the U6RNA internal control.

### Statistical analysis

Statistical analysis was performed using SPSS 16.0 software (SPSS, Inc., Chicago, IL, USA). Correlations were analyzed using Pearson’s correlation. The statistical significance of the differences in data was determined using the Student’s t-test, and data are expressed as the mean ± standard deviation from at least three independent experiments. Differences were considered statistically significant when P<0.05.

## Results

### Characteristics of study subjects

The demographics of the study subjects are summarized in Table I. The gender distribution between the two groups of study subjects was similar. However, healthy controls were on average younger than patients with HCC. Twenty of the 25 patients with HCC were hepatitis B surface antigen-positive, indicating concurrent HBV infection, whereas none of the healthy controls carried HBV.

### miR-101 is downregulated in human HBV-related HCC tissues

**Figure 1:**
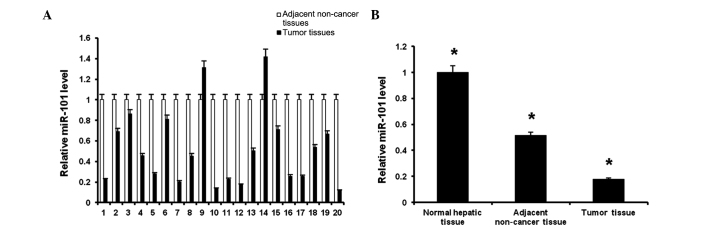
Dysregulation of miR-101 expression in human HBV-related HCC tissue compared with normal adjacent tissue and normal tissue from non-HCC patients. (A) miR-101 expression in tumor tissue compared with adjacent hepatic normal tissue for 20 HBV-related HCC patients, as determined by qPCR. (B) Average expression of miR-101 in tumor and adjacent normal tissue from HBV-related HCC patients, and in tissue from healthy controls subjects. Data are presented as the mean ± SD from three experimental repeats. ^*^P<0.05 compared with the control group. miR-101, microRNA-101; HBV, hepatitis B virus; HCC, hepatocellular carcinoma; qPCR, real-time quantitative reverse transcription-polymerase chain reaction.

To verify whether miR-101 is differentially expressed in HBV-related HCC tumors, we measured miR-101 expression levels in the 20 human HBV-related HCC tissues and adjacent noncancerous hepatic tissues by qPCR. Among the 20 matched samples, miR-101 expression within the same patient was significantly decreased in 90% of the HCC samples compared with adjacent noncancerous hepatic tissues (Fig. 1A), verifying our previous findings (20) that transformed HBV-infected HCC cells have downregulated miR-101 expression.

On average, the miR-101 expression levels in HBV-related HCC tissue were ~50% lower compared with those in normal adjacent tissue (Fig. 1B, first two bars). In addition, the levels of miR-101 in both the tumor and adjacent normal tissues from human HBV-related HCC patients were downregulated compared with those in tissue from the healthy controls (Fig. 1B, third bar). These results confirm the findings in Fig. 1A and indicate that reduced tissue miR-101 levels are observed in all hepatic tissues from HCC patients, but are more marked in tumor tissue.

### miR-101 is upregulated in human HBV-related HCC serum

**Figure 2:**
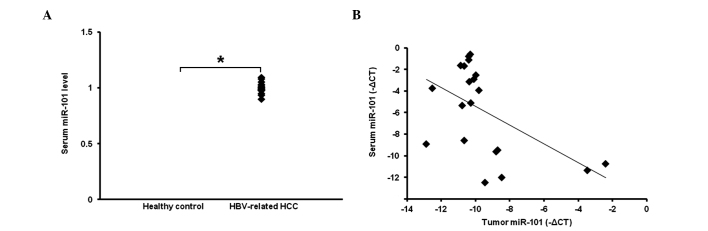
Serum miR-101 levels correlate with HBV-related HCC tissues and are upregulated in human HBV-related HCC serum. (A) Upregulated serum miR-101 expression in patients with HBV-related HCC compared with healthy controls by qPCR. The expression of miR-101 (log10 scale on the y-axis) was normalized to U6 with respect to the specimen. (B) Correlation between the miR-101 expression level (-ΔCt) in tumor tissue and serum. Statistical analysis to evaluate correlation was performed using Pearson’s correlation analysis. Data shown are the averages of three experimental repeats. ^*^P<0.05 compared with the control group. HBV, hepatitis B virus; HCC, hepatocellular carcinoma; miR-101, microRNA-101; qPCR, real-time quantitative reverse transcription-polymerase chain reaction.

Circulating miRNAs have emerged as candidate diagnostic markers for various types of cancer (14–19). We hypothesized that dysregulated miR-101 in serum may be suitable for use as a biomarker for HCC diagnosis. To test this, we assessed whether serum miR-101 levels show a similar decrease to tissue miR-101 levels in HCC patients. Notably, although miR-101 tissue levels were downregulated approximately two- to five-fold in HBV-related HCC patients in this study (Fig. 1) and our previous study (20), the expression of miR-101 in HBV-related HCC serum increased by approximately 10-fold compared with the healthy controls (Fig. 2A). This discrepancy between the trends of downregulation in tissue from HCC patients versus upregulation in serum from HCC patients may be explained by the increased amount of overall hepatic tissue in HCC patients with tumor masses. The miR-101 levels in the tissue samples were standardized to the U6 RNA levels within the tissue, and therefore should account for cell number; however, the serum levels assess absolute circulating miR-101 levels and are standardized to the higher level of U6 RNA in serum, which may arise from a variety of cell sources. An alternative explanation for this discrepancy is that different mechanisms may regulate the production of miRNA within the cell and its release. Regardless of the explanation, the marked increase in serum levels of miR-101 may be suitable for use as a diagnostic biomarker for HBV-related HCC.

### Inverse correlation between serum miR-101 levels and HBV-related HCC tissue miR-101 levels

To verify the disparate results for tissue and serum miR-101 expression trends, we assessed whether serum miR-101 levels have a positive correlation with miR-101 levels in HBV-related HCC tissue. miR-101 mRNA expression levels in HBV-related HCC tumor tissue and serum were compared using the qPCR data for all 20 HCC patients. Our results show that serum miR-101 levels have an inverse correlation with the levels in tumor tissue (Fig. 2B). This verifies that different mechanisms underlie the trends for the downregulation of miR-101 in tissue and the upregulation of miR-101 in serum of HCC patients.

### miR-101 expression and clinicopathological characteristics

To determine whether circulating miR-101 levels are indicative of the state of HCC progression, we studied whether miR-101 serum expression correlated with the clinicopathological characteristics of HCC. miR-101 levels were not statistically associated with patient age or gender, or several of the classic markers for HCC progression, including AFP levels, alanine transaminase, aspartate aminotransferase, cirrhosis and tumor-node-metastasis staging; however, a statistically significant correlation was observed between miR-101 expression and HBsAg, HBV DNA level and tumor size (Table II). This significant correlation may indicate that miR-101 expression is important in HBV-related HCC tumorigenesis and may be a potential tumor marker for this type of cancer when it is associated with HBV positivity, as is the case for ~80% of all HCCs (2).

## Discussion

HCC, which is the fifth most common cause of cancer worldwide, has an extremely poor prognosis. The early diagnosis of HCC is of great clinical importance and may improve the prognosis of HCC if patients were to receive surgical treatment early. Despite noteworthy advances in the effort to develop noninvasive serum biomarkers for the diagnosis of HCC, the reliability of biomarkers, such as AFP, remains debatable. Indeed, the specificity of AFP is low, particularly in the context of chronic liver disease (21). Accordingly, novel biomarkers for early HCC diagnosis are urgently required.

miRNAs have been identified in many body fluids, including serum and plasma (22), and studies have indicated circulating miRNAs as potential biomarkers for several disease conditions, including human cancer (23–25). Circulating miRNAs can therefore be considered representative of certain pathological conditions. Moreover, their accessibility and high stability in the circulatory system (15) make them ideal biomarkers, particularly for the surveillance of early-stage, pre-symptomatic diseases in at-risk patients (26). Studies have shown that miRNAs can function as oncogenes or tumor suppressors to promote or prevent HCC development (27,28). Therefore, it is anticipated that circulating miRNAs are also affected during HCC progression. A previous study reported altered levels of circulating miRNAs in association with HCC. For instance, the serum level of miR-122 was shown to be higher in HCC patients than in healthy controls and to be reduced in post-operative serum samples (29). Although the clinical significance of these findings has not been elucidated in detail, these findings demonstrate that circulating miRNAs may be noninvasive diagnostic or prognostic markers for HCC.

In this study, we demonstrated that miR-101 was downregulated in 90% of HBV-positive HCC tumor tissues compared with adjacent noncancerous tissue. Furthermore, these miR-101 levels were decreased compared with those in healthy controls. Apparently contradictory to these findings, we found that the serum miR-101 expression in patients with HBV-related HCC was significantly higher than that in the healthy controls. Serum miR-101 levels correlated with HBsAg, HBV DNA level and tumor size, indicating that serum miR-101 may be used as a potential predictor of HCC prognosis.

Although our results gave a high positive predictive value for our HCC cohort, there are several limitations to this study. For example, the post-surgical serum samples were small in size and varied in time point. It is imperative to test more longitudinal samples in order to justify the specific time or period at which the circulating miRNAs return to basal levels.

Substantial evidence has implicated that serum-based miRNAs are useful as noninvasive biomarkers for different types of cancer (30–33); however, little is known regarding the source of circulating miRNAs and the mechanisms that control their biogenesis. It is speculated that miRNAs may enter the circulation via secretion from blood cells or tissues/cells that are affected by disease (15). At present, aberrant serum miRNA levels in cancer are considered to be due to excessive secretion by primary cancer cells (34–36). Further studies are required to determine the exact time during cancer progression at which circulating miRNAs become detectable in the bloodstream.

In conclusion, our findings indicate that the fluctuation in circulating miRNAs during HCC provides an innovative approach that offers a sensitive and convenient means for the early detection of HBV-related HCC carcinogenesis. Serum miR-101 expression, which was closely associated with tumoral size in this study, provides a promising biochemical marker of HBV-related HCC.

## Figures and Tables

**Table I:** Demographics of healthy controls and patients with primary HCC.

	Healthy controls (n=20)		Patients with HCC (n=25)	
		
	No.	(%)	No.	(%)
Gender				
Male	13	65.0	19	76.0
Female	7	35.0	6	24.0
Age (years)				
≤40	17	85.0	4	16.0
41–50	2	10.0	8	32.0
51–60	1	5.0	10	40.0
>60	0	0.0	3	12.0
HBV status				
HBsAg^+^	0	0.0	20	80.0
HBsAg^−^	20	100.0	5	20.0

HCC, hepatocellular carcinoma; HBV, hepatitis B virus; HBsAg, hepatitis B surface antigen.

**Table II:** Clinicopathological features and miR-101 expression in HCC.

Clinicopathological features	No. (n=25)	miR-101 expression (−ΔCt)		P-value

		<10.13	≥10.13	
Gender				
Male	19	10	9	0.409
Female	6	2	4	
Age (years)				
<40	4	1	3	0.315
≥40	21	11	10	
HBsAg				
+	19	12	7	0.047
−	6	1	5	
HBV DNA				
<1.0×10e^3^	10	9	1	0.001
≥1.0×10e^3^	15	3	12	
AFP (μg/l)				
<400	18	8	10	0.568
≥400	7	4	3	
ALT (U/l)				
<40	9	2	7	0.053
≥40	16	10	6	
AST (U/l)				
<40	11	5	6	0.821
≥40	14	7	7	
Tumor size (cm)				
<5	16	11	5	0.006
≥5	9	1	8	
Cirrhosis				
+	18	9	9	0.748
−	7	3	4	
TNM staging				
I	6	5	1	0.137
II	14	5	9	
III	5	3	2	

miR-101, microRNA-101; HCC, hepatocellular carcinoma; HBsAg, hepatitis B surface antigen; HBV, hepatitis B virus; AFP, α-fetoprotein; ALT, alanine transaminase; AST, aspartate aminotransferase; TNM, tumor-node-metastasis.

## Abbreviations

miR-101: microRNA-101
HBV: hepatitis B virus
HBsAg: hepatitis B surface antigen
HCC: hepatocellular carcinoma
AFP: α-fetoprotein
AST: aspartate aminotransferase
ALT: alanine transaminase
